# Efficacy of Immune Checkpoint Inhibitors in Patients with Mismatch Repair-Deficient or Microsatellite Instability-High Metastatic Colorectal Cancer: Analysis of Three Phase-II Trials

**DOI:** 10.7759/cureus.19893

**Published:** 2021-11-25

**Authors:** Luca Cancanelli, Melania Rivano, Lorenzo Di Spazio, Marco Chiumente, Daniele Mengato, Andrea Messori

**Affiliations:** 1 Hospital Pharmacy Department, Azienda Unità Locale Socio Sanitaria (ULSS) 2 Marca Trevigiana, Treviso, ITA; 2 Clinical Oncology Pharmacy Department, Armando (A) Businco Hospital, Cagliari, ITA; 3 Hospital Pharmacy Department, Santa (S) Chiara Hospital, Trento, ITA; 4 Scientific Direction, Italian Society for Clinical Pharmacy and Therapeutics, Milano, ITA; 5 Hospital Pharmacy Department, Azienda Ospedaliera Universitaria di Padova, Padova, ITA; 6 Health Technology Assessment (HTA) Unit, Regione Toscana, Firenze, ITA

**Keywords:** meta-analysis, reconstruction of patient-level data, kaplan-meier survival curves, individual-patient data, immune checkpoint inhibitors, microsatellite instability, metastatic colorectal cancer

## Abstract

Programmed cell death ligand 1 (PD-L1) and programmed cell death protein 1 (PD-1) inhibitors are increasingly used in a variety of solid tumors. In patients with DNA mismatch repair-deficient (dMMR)/microsatellite instability-high (MSI-H) metastatic colorectal cancer, their efficacy has been demonstrated in recently published phase-II trials. However, an indirect comparison of effectiveness between pembrolizumab, nivolumab, and nivolumab+ipilimumab is not yet available.

After a standard literature search, we analyzed four overall survival (OS) curves from three phase-II trials. Individual patient data were reconstructed from each curve using a specific web-based technique (Shiny method). Indirect statistical comparisons were made based on hazard ratio (HR) and restricted mean survival time (RMST).

Nivolumab+ipilumumab had a better HR compared with pembrolizumab (0.65, 95% confidence interval [CI], 0.43 to 1.002, p=0.051); the difference being close to statistical significance. In the analysis based on RMST, the combination of nivolumab+ipilimumab showed a significantly longer OS than pembrolizumab (improvement in RMST, 1.08 mos; 95%CI, 0.11 to 2.06; p=0.029). The other two pairwise differences in RMST (nivolumab vs. pembrolizumab and nivolumab+ ipilimumab vs. nivolumab) had a smaller magnitude (0.25 mos, 95%CI, -0.99 to 1.48, and 0.84 mos, 95%CI, -0.40 to 2.07, respectively) and were far from statistical significance.

Our results favoring the combination of nivolumab+ipilimumab in metastatic colorectal cancer must be viewed with caution owing to the indirect nature of our statistical comparisons. With this limitation in mind, the magnitude of the incremental benefit for the above combination treatment was estimated to be around one month over a follow-up of 15 months.

## Introduction and background

Colorectal cancer (CRC) is the fifth leading cause of cancer-related death, with an estimated 576,858 deaths worldwide [1]. It is clinically defined by its tissue of origin in the colon or rectum but is mainly a heterogeneous disease classified by its genetics [2-4]. Patients with DNA mismatch repair-deficient (dMMR)/microsatellite instability-high (MSI-H) metastatic CRC are closely associated with a mutation in the BRAF gene and benefit to a lesser degree from conventional chemotherapy [5-6]. Despite well-known genetic differences in the disease, patients with newly diagnosed metastatic CRC are generally treated with fluorouracil-based chemotherapy combined with agents targeting angiogenesis or the epidermal growth factor receptor [7]. MSI/dMMR tumors are highly infiltrated by immune cells [8] and are associated with an upregulation of checkpoint inhibitors that exhausts intratumoral cytotoxic T lymphocytes and consequently protects MSI cancer cells from their hostile immune microenvironment [8-9].

Recent evidence has shown that MSI-H/dMMR tumors achieve durable responses to single-agent programmed death 1 (PD-1) blockade or to combination regimens that include cytotoxic T-lymphocyte-associated antigen-4 inhibitor. The results of several phase II studies have demonstrated the efficacy of immune checkpoint inhibitors (ICIs) in pretreated patients with MSI/dMMR metastatic CRC [10-13]. Further trials are currently ongoing to assess ICI efficacy in first-line, adjuvant, or even neoadjuvant settings [14]. Hence, anti-PD-1 ICIs have nowadays become the new standard of care as second or subsequent line treatment of metastatic CRC. Furthermore, results from ongoing trials will likely move them to the standard of care in earlier lines of treatment but this has not yet occurred.

On the other hand, as regards the methodology for analyzing survival curves, innovative techniques (e.g. the Shiny method [15]) have recently been made available that permit to reliably reconstruct individual patient data through an automated analysis of Kaplan-Meier curves [15-17]. These techniques, which essentially belong to the field of artificial intelligence, require the availability of the following three pieces of information for each curve: 1) the Kaplan Meier graph; 2) the total number of patients included in the curve; 3) the total number of events. When these requirements are met, the performance of these techniques in reconstructing patient-level data is excellent [15]. While the Shiny method efficiently reconstructs individual survival times, it should be kept in mind that this is a univariate analysis. As a result, unless the unlikely hypothesis in which one or more covariates are separately presented in additional Kaplan-Meier curves, the Shiny method is unable to test the effect of covariates on survival according to multivariate statistics. Only the primary time-to-event end-point (e.g. survival) can, in fact, be assessed through a univariate design.

In this context, the objective of the present review was to examine the most recent trials that have studied ICIs in patients with MSI-H/dMMR metastatic CRC and to apply the above-mentioned techniques of patient-data reconstruction to perform indirect comparisons across treatments without using any meta-analysis statistics.

## Review

Our review consisted of four main phases: a) literature search; b) reconstruction of individual patient data; c) statistical analysis of reconstructed survival curves; d) interpretation of survival data. All survival statistics were performed under the R-platform [18].

Literature search

We carried out a literature search to identify the clinical studies eligible for our analysis. Our search was conducted in PubMed (last query on October 10, 2021) and covered the period from January 2010 to the present date. The search string "((colorectal neoplasm’s[MeSH Terms]) AND (microsatellite instability[MeSH Terms])) AND ((pembrolizumab) OR (nivolumab) OR (atezolizumab) OR (ipilimumab) OR (dostarlimab) OR (durvalumab) OR (avelumab)))" was employed in combination with the filter “clinical trial”. We also searched through the Cochrane Library and the ClinicalTrials.gov database.

Our review was designed to evaluate clinical trials that met the following criteria: a) patients with MSI/dMMR metastatic CRC; b) phase-II or phase-III; c) chemoimmunotherapy given in single-arm phase-II trials or as the experimental arm in phase-III trials; d) determination of overall survival (OS) based on follow-up of at least 15 months; e) availability of a Kaplan-Meier curve on OS.

**Figure 1:**
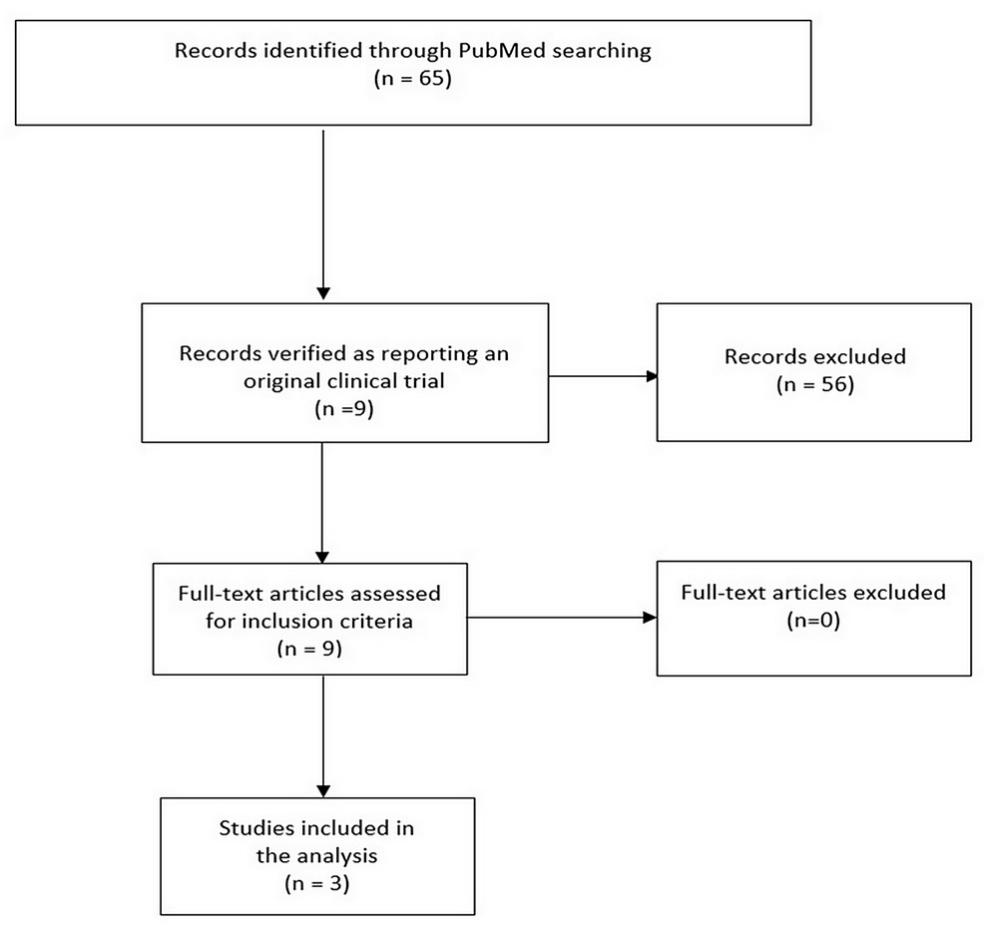
PRISMA Flow Diagram PRISMA: Preferred Reporting Items for Systematic Reviews

Our PubMed search extracted a total of 65 eligible papers. After excluding the papers that did not report OS, we eliminated duplicate entries and finally identified three trials that met our inclusion criteria [11-13]. The Preferred Reporting Items for Systematic Reviews (PRISMA) workflow is shown in Figure 1.

In the KEYNOTE-164 phase II study evaluating pembrolizumab, patients from cohort A had received two prior lines of standard therapy while cohort B had received one prior line of standard therapy [11]. In the CHECKMATE-142 phase II study, nivolumab was given to previously treated patients who had received in the majority of cases three lines of treatment [12]. Finally, in the third included trial, patients treated with nivolumab plus ipilimumab had received at least two prior lines of therapy [13]. The study on avelumab could not be analyzed because it did not report an OS curve [19].

Reconstruction of individual patient data

After the selection of included trials, each of the Kaplan-Meier OS curves was analyzed according to the following procedure. First, the graph was digitalized and converted into x-y data pairs using Webplotdigitizer [17]. Then, the Shiny package (Version: 1.2.2.0; subprogram “Reconstruct Individual Patient Data”; https://www.trialdesign.org/one-page-shell.html#IPDfromKM [15]) was used to reconstruct patient-level data on the basis of x-y data pairs of the curve, the total number of enrolled patients, and the total number of events. After reconstructing each curve, individual patient data from the three trials were subjected to statistical analysis of OS.

Statistical analysis of reconstructed survival curves

After reconstructing each curve, the individual patient data were first analyzed to generate the Kaplan-Meier curves for each treatment and to compare them through Cox statistics and hazard ratio (HR). For this purpose, three packages (“coxph”, “survfit”, and “ggsurvplot”) were used under the R-platform [18]. Furthermore, another R-package ("survRM2”) was run to determine the value of restricted mean survival time (RMST) along with its 95% confidence interval (CI). These estimations of RMST employed a milestone of 15 months, which represented the longest follow-up reached by all included cohorts. To extend the survival analysis over a lifetime horizon, we also determined the mean lifetime survival (MLS) from the same data employed to determine RMST; MLS was modeled according to the Weibull distribution managed under the R-platform (“eha” package [18]).

**Figure 2:**
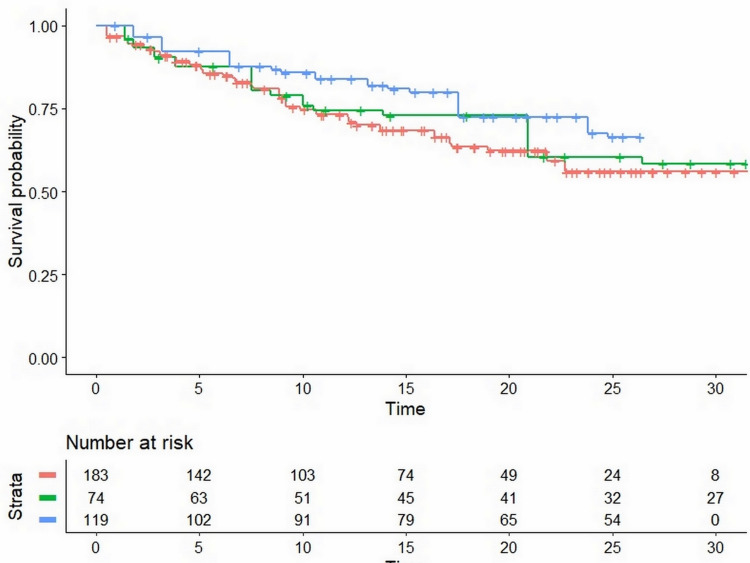
Kaplan-Meier curves from reconstructed patient-level data Pooled Kaplan-Meier survival curves obtained by the reconstruction of individual patient data from three trials (KEYNOTE-164; CHECKMATE-142; Overman et al.). See text for details. Treatment groups: pembrolizumab (KEYNOTE-164) in red; nivolumab (CHECKMATE-142) in green; nivolumab+ipilimumab (Overman et al.) in blue. Time expressed in months.

Figure 2 shows the Kaplan-Meier curves generated from the reconstructed patient-level data of the three phase-II trials. 

The statistical analysis of between-treatment differences yielded the values of HR shown in Table 1. Noteworthy, nivolumab+ipilimumab had a better HR compared with pembrolizumab, the difference being close to statistical significance. Survival in the two cohorts of the CHECKMATE-142 study is also reported in Appendix A. The values of RMST estimated from the three trials are shown in Table 1, along with the values of MLS.

**Table 1:** Summary of the three clinical studies included in the analysis Notes: values of RMST refer to a milestone of 15 mos while values of HR represent a comparison with KEYNOTE-164. Abbreviations: n, number of events; N, number of patients; HR, hazard ratio; RMST, restricted mean survival time; CI, confidence interval; mos, months; MLS, mean lifetime survival

Cohort	Reference	Treatment	No. of patients (n/N)	Median OS (mos)	HR	RMST with 95%CI (mos)	MLS with 95%CI (mos)
KEYNOTE-164 – cohort A	Le et al. [11]	Pembrolizumab	19/61	20.9	1	12.3 (11.6 to 13.0)	53.6 (40.1 to 71.6)
KEYNOTE-164 – cohort B			19/63				
CHECKMATE-142	Overman et al. [12]	Nivolumab	53/74	22.7	0.86 (0.54 to 1.36, p=0.52)	12.6 (11.5 to 13.6)	55.3 (42.8 to 71.5)
Nivolumab+ipilimumab	Overman et al. [13]	Nivolumab+ ipilimumab	86/119	Not reached	0.65 (0.43 to 1.002, p=0.051)	13.4 (12.7 to 14.1)	61.4 (43.4 to 86.7)

Interpretation of the survival data

The combination of nivolumab+ipilimumab showed better OS than monotherapy with pembrolizumab according to both HR and RMST. In the first case, the difference was at limits of statistical significance (p=0.051) while in the second, the difference achieved significance (p=0.029). According to the RMST, the prolongation in OS had a magnitude of 1.08 months (95% confidence interval (CI), 0.11 to 2.06). It should be kept in mind that the RMST was restricted at the milestone of 15 months. Interestingly enough, the values of MLS shown in Table 1 indicate that the incremental benefit is much longer (7.8 months) if extrapolated over a lifetime horizon.

The other two pairwise differences in RMST (nivolumab vs. pembrolizumab and nivolumab+ipilimumab vs. nivolumab) had a smaller magnitude (0.25 months, 95%CI, -0.99 to 1.48, and 0.84 months, 95%CI, -0.40 to 2.07, respectively) and remained far from statistical significance.

Variability in MLS was wide owing to the extrapolated nature of this parameter. It was instead very limited for RMST, likely because the follow-up of these patients (and consequently the milestone) was limited to 15 months.

These results concerning pembrolizumab and nivolumab alone or in combination with ipilimumab in previously treated patients with MSI/dMMR metastatic CRC have led to the approval of these treatments by the Food and Drug Administration (FDA) in the US. Also, the study by Andrè et al., conducted in patients with the same disease condition but not previously treated, has contributed to this decision [20].

A certain heterogeneity across these patient cohorts emerged from our analysis. In particular, different numbers of previous treatment lines were employed in the three trials. On the other hand, although there were no substantial differences in the baseline characteristics among the trials, these characteristics, in most cases, did not include some important prognostic factors. For example, the BRAF-V600E mutation, which is found in 8%-10% of metastatic colorectal cancer patients, is recognized to be a poor prognostic factor [21]. Likewise, EGFR mutant tumors have a generally low response to immune checkpoint inhibitors, although this has been demonstrated especially in non-small-cell lung cancer [22-23]. Hence, one cannot exclude the hypothesis that unbalances in disease burden or prognostic factors in study arms might explain the different outcomes or the lack of differences. Keeping this point in mind, the difference in effectiveness among treatments is a potential explanation for the findings emerging from our analysis. This difference could have an important impact on the selection of ICIs in clinical practice. It should, however, be stressed that our comparisons had an indirect nature, and the consequences of this limitation are well-known.

Several randomized trials are currently ongoing to evaluate the efficacy of ICIs versus standard-of-care chemotherapy ± targeted therapy in a first- or second-line metastatic setting (see Appendix B). To date, only partial results of the KEYNOTE 177 study are available. In this trial, after a median follow-up of 32.4 months, pembrolizumab was superior to chemotherapy with respect to progression-free survival (median, 16.5 vs. 8.2 months; hazard ratio, 0.60; 95%CI, 0.45 to 0.80; P=0.0002), but data on OS still remain blinded until the final analysis [24]. The other studies are in the recruitment phase and, therefore, their results are not available.

Despite the positive results of the three trials included in our analysis, a number of questions remain unanswered. First of all, no predictive biomarkers are currently validated to predict the resistance to therapy [14]. Consequently, the choice of the best treatment for these patients (monotherapy or not) remains difficult. Second, the development of adjuvant or neo-adjuvant settings is of interest and deserves further insight. All in all, while the effectiveness of ICIs in CRC is undisputed, one should also keep in mind that these agents have demonstrated remarkable effectiveness in many other malignancies, as pointed out in recent reviews [25].

## Conclusions

The results currently available concerning OS in metastatic colorectal cancer are in favor of the combination of nivolumab+ipilimumab. However, this evidence must be viewed with caution owing to the indirect nature of the statistical comparisons reported above. With this limitation in mind, the magnitude of the incremental benefit for the above combination treatment was estimated to be around one month over a follow-up of 15 months and approached eight months over a lifetime perspective.

## Figures and Tables

**Figure 3:**
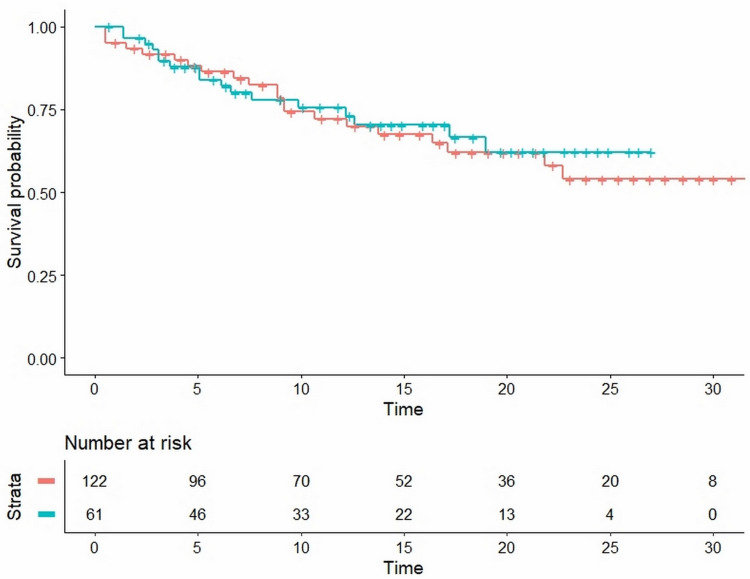
Secondary analysis: comparison of cohort A (in red) vs cohort B (in blue) in the KEYNOTE-164 trial The results obtained from reconstructed patient-level data (HR, 0.89; 95%CI, 0.51 to 1.56; p=0.683) are virtually identical to those reported in the original trial. Time expressed in months. Abbreviations: HR, hazard ratio; CI, confidence interval

## Appendices

Appendix A

Appendix B 

**Table 2:** Ongoing studies on ICIs in colorectal cancer ICIs: immune checkpoint inhibitors

Six active studies
KEYNOTE-177 NCT02563002 (closed to recruitment): Phase III First line Metastatic colorectal cancer Pembrolizumab (anti-PD1) VS Standard-of-care chemotherapy (mFOLFOX6 or FOLFIRI alone or in combination with bevacizumab or cetuximab).
COMMIT NCT02997228 (recruiting): Phase III First line Metastatic colorectal cancer Atezolizumab (anti-PD-L1) VS Atezolizumab + mFOLFOX6 + bevacizumab VS mFOLFOX6 + bevacizumab.
CA209-8HW NCT04008030 Phase IIIb (recruiting): Metastatic colorectal cancer Nivolumab VS Nivolumab and ipilimumab VS Investigator’s Choice chemotherapy ± targeted therapy irrespective of prior treatments.
PRODIGE 54— SAMCO NCT03186326 (recruiting): Randomized phase II Second line Metastatic colorectal cancer Avelumab (anti-PD-L1) VS Standard-of-care chemotherapy (mFOLFOX6 or FOLFIRI alone or in combination with bevacizumab or cetuximab).
Alliance A021502 NCT02912559 (recruiting): Phase III Adjuvant Stage III colon cancer Atezolizumab (anti-PDL1) + mFOLFOX6 vs mFOLFOX6.
POLEM NCT03827044 (recruiting): Phase III Adjuvant Stage III colon cancer (MSI or POLEmutated) Avelumab (anti-PDL1) + CAPOX 3 months or capecitabine 6 months VS CAPOX 3 months or capecitabine 6 months.
